# Mimicry and well known genetic friends: molecular diagnosis in an Iranian cohort of suspected Bartter syndrome and proposition of an algorithm for clinical differential diagnosis

**DOI:** 10.1186/s13023-018-0981-5

**Published:** 2019-02-13

**Authors:** Maryam Najafi, Dor Mohammad Kordi-Tamandani, Farkhondeh Behjati, Simin Sadeghi-Bojd, Zeineb Bakey, Ehsan Ghayoor Karimiani, Isabel Schüle, Anoush Azarfar, Miriam Schmidts

**Affiliations:** 1 Genome Research Division, Human Genetics department, Radboud University Medical Center, Geert Grooteplein Zuid 10, 6525KL Nijmegen, The Netherlands; 2 Departement of Biology, University of Sistan and Baluchestan, Zahedan, Iran; 3 Genetics Research Center, University of Social Welfare and Rehabilitation Sciences, Tehran, Iran; 4 Children and Adolescents Health Research Center, resistant tuberculosis institute, Zahedan University of Medical Sciences, Zahedan, Iran; 5 Razavi Cancer Research, Razavi Hospital, Imam Reza International University, Mashhad, Iran; 6 Next Generation Genetic Polyclinic, Mashhad, Iran; 7 Department of Pediatrics, Faculty of Medicine, Mashhad University of Medical Sciences, Mashhad, Iran; 8 Center for Pediatrics and Adolescent Medicine, Freiburg University Hospital, Freiburg University Faculty of Medicine, Mathildenstrasse 1, 79112 Freiburg, Germany; 9 Center for Pediatrics and Adolescent Medicine, University Hospital Freiburg, 79112 Freiburg, Germany

**Keywords:** Bartter syndrome, Whole exome sequencing, Pseudo-Bartter-syndrome

## Abstract

**Background:**

Bartter Syndrome is a rare, genetically heterogeneous, mainly autosomal recessively inherited condition characterized by hypochloremic hypokalemic metabolic alkalosis. Mutations in several genes encoding for ion channels localizing to the renal tubules including *SLC12A1, KCNJ1, BSND, CLCNKA, CLCNKB, MAGED2* and *CASR* have been identified as underlying molecular cause. No genetically defined cases have been described in the Iranian population to date. Like for other rare genetic disorders, implementation of Next Generation Sequencing (NGS) technologies has greatly facilitated genetic diagnostics and counseling over the last years. In this study, we describe the clinical, biochemical and genetic characteristics of patients from 15 Iranian families with a clinical diagnosis of Bartter Syndrome.

**Results:**

Age range of patients included in this study was 3 months to 6 years and all patients showed hypokalemic metabolic alkalosis. 3 patients additionally displayed hypercalciuria, with evidence of nephrocalcinosis in one case. Screening by Whole Exome Sequencing (WES) and long range PCR revealed that 12/17 patients (70%) had a deletion of the entire *CLCNKB* gene that was previously identified as the most common cause of Bartter Syndrome in other populations. 4/17 individuals (approximately 25% of cases) were found to suffer in fact from pseudo-Bartter syndrome resulting from congenital chloride diarrhea due to a novel homozygous mutation in the *SLC26A3* gene, Pendred syndrome due to a known homozygous mutation in *SLC26A4*, Cystic Fibrosis (CF) due to a novel mutation in *CFTR* and apparent mineralocorticoid excess syndrome due to a novel homozygous loss of function mutation in *HSD11B2* gene. 1 case (5%) remained unsolved.

**Conclusions:**

Our findings demonstrate deletion of *CLCNKB* is the most common cause of Bartter syndrome in Iranian patients and we show that age of onset of clinical symptoms as well as clinical features amongst those patients are variable. Further, using WES we were able to prove that nearly 1/4 patients in fact suffered from Pseudo-Bartter Syndrome, reversing the initial clinical diagnosis with important impact on the subsequent treatment and clinical follow up pathway. Finally, we propose an algorithm for clinical differential diagnosis of Bartter Syndrome.

**Electronic supplementary material:**

The online version of this article (10.1186/s13023-018-0981-5) contains supplementary material, which is available to authorized users.

## Background

Bartter Syndrome (BS), firstly reported by Bartter and his colleagues in 1962, is a very rare autosomal recessive salt-losing tubulopathy characterized by hypokalemic metabolic alkalosis with normotensive hyperreninemia and hyperaldosteronism [1] occurring with an estimated incidence of 1.2/million in the population [2]. Based on the loss of function mutations in the salt reabsorption transporters and channels in the thick ascending limb of the loop of Henle, genetically, five variants of this syndrome have been described: type I resulting from loss of function mutations in the Solute Carrier Family 12 Member 1 *SLC12A1* gene encoding the apical furosemide-sensitive Na-K-Cl co-transporter (OMIM #600839), type II caused by mutations in the potassium voltage-gated channel subfamily J member 1 (*KCNJ1)* gene encoding the apical renal outer medullary potassium channel (ROMK) (OMIM # 600359), type III caused by mutations in the chloride voltage-gated channel Kb (*CLCNKB)* gene encoding the basolateral chloride channel Kb (OMIM #602023), type IVa resulting from dysfunction of the Barttin CLCNK type accessory beta subunit (*BSND)* gene encoding Barttin, a subunit of chloride-channels Ka and Kb (OMIM #606412) and finally, type IVb caused by co-mutation in the *CLCNKA* and *CLCNKB* genes (OMIM #602024) [3–7]. Gitelman syndrome which shares several clinical characteristics with BS type III has been described later in history. In contrast to BS type III, Gitelman syndrome is caused by mutations in a single gene, *SLC12A3* (OMIM #263800)*,* encoding the thiazide-sensitive sodium chloride co-transporter (NCCT) in the distal convoluted tubule [8, 9]. More recently, 2 genes, namely calcium sensing receptor (*CASR)* (OMIM #601198) and MAGE family member D2 (*MAGED2)* (OMIM #300971, BS type V) have been identified which cause autosomal dominant and X-linked recessive forms of BS [10, 11].

Currently, from a phenotypical perspective, BS has been classified into three different forms according to the average age at onset of symptoms: antenatal BS, the most severe form of BS, marked by polyhydramnios, hypercalciuria, nephrocalcinosis, hypochloremia and failure to thrive in infancy; Second, classic BS which has a milder phenotype and is usually diagnosed during late adulthood. And third, Gitelman syndrome which compared to other variants is marked with hypocalciuria and hypomagnesaemia and is usually diagnosed during late childhood and adulthood. In most cases, BS IVa and IVb subtypes are accompanied by sensorineural deafness [12]. However, the BS subtype can often not be determined clinically with certainty due to similar presentation of different forms, rendering diagnostics and precise prognosis complex [13]. Despite the need for rigorous classification of BS phenotypes, currently few practical indicators exist.

Making the landscape of BS and BS-like clinical presentations appear even more complex, several renal and extra-renal disorders as congenital chloride diarrhea, Pendred syndrome, Cystic fibrosis as well as some acquired conditions associated (for example laxative abuse) may present clinically in a similar way as BS with regards to hypokalemic metabolic alkalosis. However, there is only a limited number of reports in the literature investigating misdiagnosis of BS where in fact other rare hypokalemic disorders (CF) were causative for the phenotype [14–22]. Nevertheless, such clinical misdiagnosis can result in serious health problems due to wrong treatment choices [23, 24]. However, novel high throughput sequencing technologies nowadays offer an additional diagnostic tool refining clinical diagnostics.

In the present study, we describe 17 patients from 15 Iranian families with a clinical diagnosis of BS. Implementing WES as additional diagnostic step combined with long-range PCR screening for *CLCNKB*, we identified the underlying genetic cause in 16/17 cases. While we confirmed the clinical diagnosis of BS in 12 cases, our genetic analysis established a diagnosis different from BS in 4 cases. Additionally, we propose a cost-efficient clinical differential diagnostic algorithm.

## Results

Clinical and genetics findings are summarized in Table 1. Laboratory results of all 17 patients showed severe hypokalemic alkalosis. Comparison of normal ranges of urinary calcium/creatinine ratio for age (hypercalciuria screening) indicated that case 3 showed hypocalciuria and case 2, 8, and 10 showed hypercalciuria (age dependent normal urinary creatinine/calcium ratios are shown in Table S2). 9 out of 17 cases clinically presented before age of 1 year, 2 out of 17 cases between 1 to 2 years old, 5 out of 17 cases between 2 to 3 years old and one case at the age of 6 years. Case 9 and case 15 did not survive due to severe hypokalemic metabolic alkalosis. In summary, we observed a spectrum of phenotypes ranging from BS type I to Gitelman syndrome in these families.

**Table 1:** Clinical characteristics and description of the genetic findings in the cohort

Code	sex	weight	Hight	Age	Cr(mg/dL) (nl 0.6–1.0)	Na mEq/L (nl 135–155)	K mEq/L (nl 3.5–5.3)	Ca mg/dL (nl 8.6–10.2)	PH (nl 7.35–7.45)	HCO3 mEq/L (nl 22–26)	UrineCa/Cr > 0.2	Defective gene	variant	Mode of detection	Final diagnosis
1	F	4.5	60	0.5	0.5	126	2.4	NA	7.61	31	0.9	CLCNKB	c.(?_-1)_(*1_?) del, p.0	WES	Bartter syndrome
2	F	3.7	65	0.5	1	124	2.2	10	7.57	48	3.5	CLCNKB	c.(?_-1)_(*1_?) del, p.0	PCR	Bartter syndrome
3	F	5.1	64	0.5	0.6	125	2.7	NA	7.58	37	0.06	CLCNKB	c.(?_-1)_(*1_?) del, p.0	PCR	Bartter syndrome
4	M	4.7	60	0.6	0.5	144	2.1	NA	7.66	35	0.1	CLCNKB	c.(?_-1)_(*1_?) del, p.0	WES	Bartter syndrome
5	M	10	96	6	0.7	144	2	8	7.56	32	0.5	?	–	WES	–
6	M	9	83	3	0.5	131	2.8	10	7.47	31	0.8	CLCNKB	c.(?_-1)_(*1_?) del, p.0	PCR	Bartter syndrome
7	F	8.35	85	2.5	0.6	123	2.5	8	7.51	32	4.25	CLCNKB	c.(?_-1)_(*1_?) del, p.0	WES	Bartter syndrome
8	F	7.4	76	2.5	0.5	125	2.1	7	7.65	39	4.75	CLCNKB	c.(?_-1)_(*1_?)del, p.0	PCR	Bartter syndrome
9	M	3.2	63	0.5	0.5	134	3.4	NA	7.47	32	NA	CLCNKB	c.(?_-1)_(*1_?)del, p.0	PCR	Bartter syndrome
10	F	4.6	60	0.9	0.5	138	3.2	10.7	7.52	27	3.6	CLCNKB	c.(?_-1)_(*1_?) del, p.0	PCR	Bartter syndrome
11	M	5.7	75	0.4	0.5	134	3	NA	7.47	27	NA	CLCNKB	c.(?_-1)_(*1_?)del, p.0	PCR	Bartter syndrome
12	F	7.25	60	3	1	135	2.4	NA	7.53	31	0.88	CLCNKB	c.(?_-1)_(*1_?) del, p.0	WES	Bartter syndrome
13	M	5.2	67	1.3	0.4	126	2.5	8.2	7.56	32	0.54	CLCNKB	c.(?_-1)_(*1_?) del, p.0	WES	Bartter syndrome
14	M	7	73	2.3	0.6	132	2.1	7	7.58	34	0.55	CFTR	c.473G > A, p. (Ser158Asn)	WES	Cystic Fibrosis
15	F	2.9	48	0.3	1	131	2.6	9.5	7.56	47	NA	SLC26A3	c.971 + 1G > T, p.?	WES	Conjenital chloride diarrea
16	M	NA	53	1.6	0.4	140	3	10.2	7.5	30.5	0.5	SLC26A4	c.1226G > A, p. (Arg409His)	WES	Pendred syndrome
17	M	5.5	NA	0.6	0.5	132	2.6	1.50	7.50	31.2	NA	HSD11B2	c.1120C > T, p.(Arg374*)	WES	Apparent mineralocorticoid excess

**Fig. 1:**
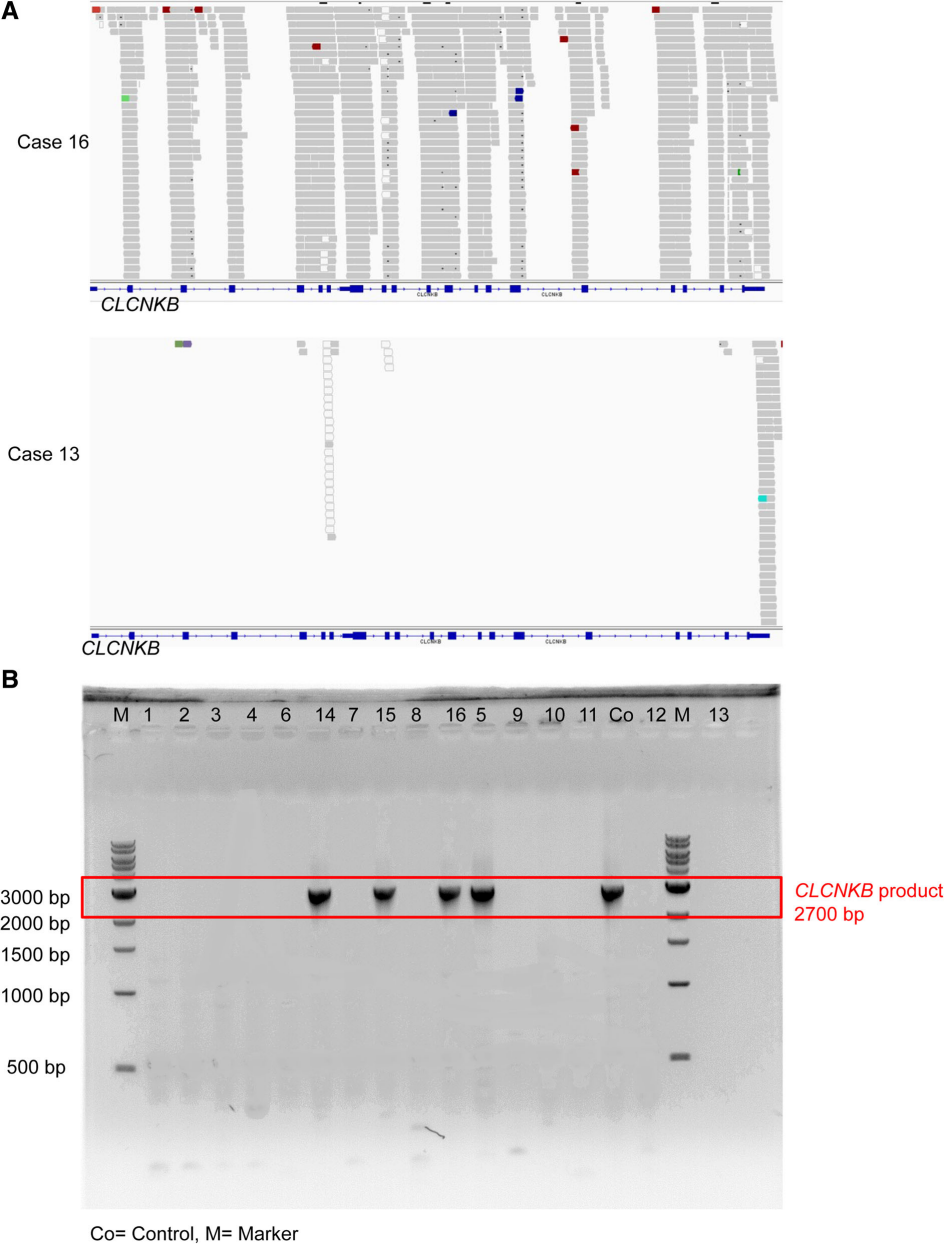
Visualization of *CLCNKB* deletion detected. (**a**) BAM files generated from WES data and displayed in IGV visualizing the deletion of the entire *CLCNKB* gene in case 13 (bottom) while reads are present in case 16 (top) who carries a homozygous mutation in *SLC26A4*. (**b**) Confirmation of *CLCNKB* deletion by long range PCR shown as absence of *CLCNKB* product in 12/16 patients on a 1% agarose gel containing ethidium bromide. M = marker, Co = control

In order to investigate the underlying genetic causes, we proceeded with WES analysis in patient 13 as an index case. This revealed a deletion of the entire *CLCNKB* gene (Fig. 1a). We therefore proceeded to check the remaining 16 patients by Sanger sequencing for this deletion. Due to high sequence similarity between *CLCNKA* and *CLCNKB*, we used long range PCR generating a *CLCNKB* specific gene product by using primer pairs with the forward primer in exon 9 and the reverse primer in exon 14. This confirmed a homozygous *CLCNKB* in 11 out of the remaining 16 cases as well as the index case sent for WES initially. A total of 12 out of the 17 cases were found to carry the deletion (Fig. 1b).

The remaining 5 samples not found to carry the CLCNKB deletion were subsequently sent for WES. This revealed a novel homozygous *CFTR* missense mutation (NM_000492.3 (*CFTR*): c.473G > A, p. (Ser158Asn)) in case 14. The mutated amino acid is highly conserved in 10 species down to zebrafish (Additional file 1: Figure S1) and the variant minor allele frequency is extremely low in control samples (0.00007381 in genomAD). Further, a different variant at the same position, *CFTR* c.473G > C has been previously reported in a case of Cystic fibrosis [25]. The patient was a 27 months old boy with a clinical BS diagnosis living in the southeast of Iran where a hot and dry weather pattern is prevalent. His medical records revealed parental consanguinity and history of failure to thrive, delayed growth, muscle weakness, dehydration and polyuria. The diagnosis of BS had been established based on the electrolytes disturbance (K^+^: 2.1 mEq/L), and blood gas analysis (HCO_3_^−^: 34 mEq/L, PH: 7.58). Treatment included KCl per 10 mEq/day and he had not been investigated for CF due to lack of significant respiratory symptoms.

WES identified a novel homozygous canonical splice site mutation at the exon /intron boundary of exon 8 of the *SLC26A3* gene (NM000111.2; c.971 + 1G > T) for case 15. Recessive loss of function mutations in *SLC26A3* have been previously reported to cause congenital chloride diarrhea [26]. This case was a 3 months old girl with a history of polyhydramnios, failure to thrive, dehydration and polyuria. At the time of birth, her body weight was 2.9 kg, height was 48 cm, and head circumference was 33 cm. After 3 months, she was hospitalized for delayed growth, muscle weakness and significant electrolyte imbalances. The diagnosis of BS had been established based on the electrolyte disturbance (K^+^: 2, 6 mEq/L), blood gas analysis (HCO_3_^−^:47, PH: 7, 56) and she did not have a noted history of diarrhea. The treatment involved KCL ampoules (10 mEq/day) under which she clinically improved.

For case 16, we detected a known homozygous missense mutation in the *SLC26A4* gene by WES (NM_000441.1 (*SLC26A4*): c.1226G > A, p. (Arg409His), and rs111033305). This variant has been previously described as pathogenic in ClinVar (RCV000169222.1). The variant had previously been found in multiple Pendred patients, however not associated with hypokalemic metabolic alkalosis [27–29]. Our case was an 18 months old boy descending from consanguineous parents with a prenatal history of severe polyhydramnios, fetal distress and meconium in the amniotic fluid. Postnatally, failure to thrive with metabolic alkalosis, vomiting and elevated body temperature were noted. Besides, results of auditory brainstem response, the auditory steady-state response, oto-acoustic emission and tympanometry tests showed bilateral mild hearing loss. The diagnosis of BS had been established based on the electrolytes disturbance (Cl^−^: 24 mEq/L, K^+^: 3 mEq/L), blood gas analysis (HCO_3_^−^: 30.5, PH: 7.50) and sensorial deafness. The stool exam showed no OVA, cyst and amoeba. The treatment involved KCl ampoules (10 mEq/day) and Spironolactone 25 mg every 8 h.

Finally, WES revealed a novel homozygous loss of function mutation in *HSD11B2* (NM_000196 (*HSD11B2*): c.1120C > T, p.(Arg374*)), establishing the genetic diagnosis of apparent mineralocordicoid excess (AME). Our case was a 6 months old boy of consanguineous parents. At the time of examination his body weight was 5.50 kg with biography of failure to thrive, hypokalemic metabolic alkalosis, and also small stones in kidney sonography. Abdominal ultrasound did not reveal any abnormalities in the liver, pancreas, spleen and bladder. His blood pressure values were in normal range in the follow up exmaminations. The diagnosis of BS was suggested based on blood electrolytes disturbance (K^+^: 2.6 mEq/L) and blood gas analysis (HCO_3_^−^: 31.2, PH: 7.50).

Cases 5 remained unsolved.

**Fig. 2:**
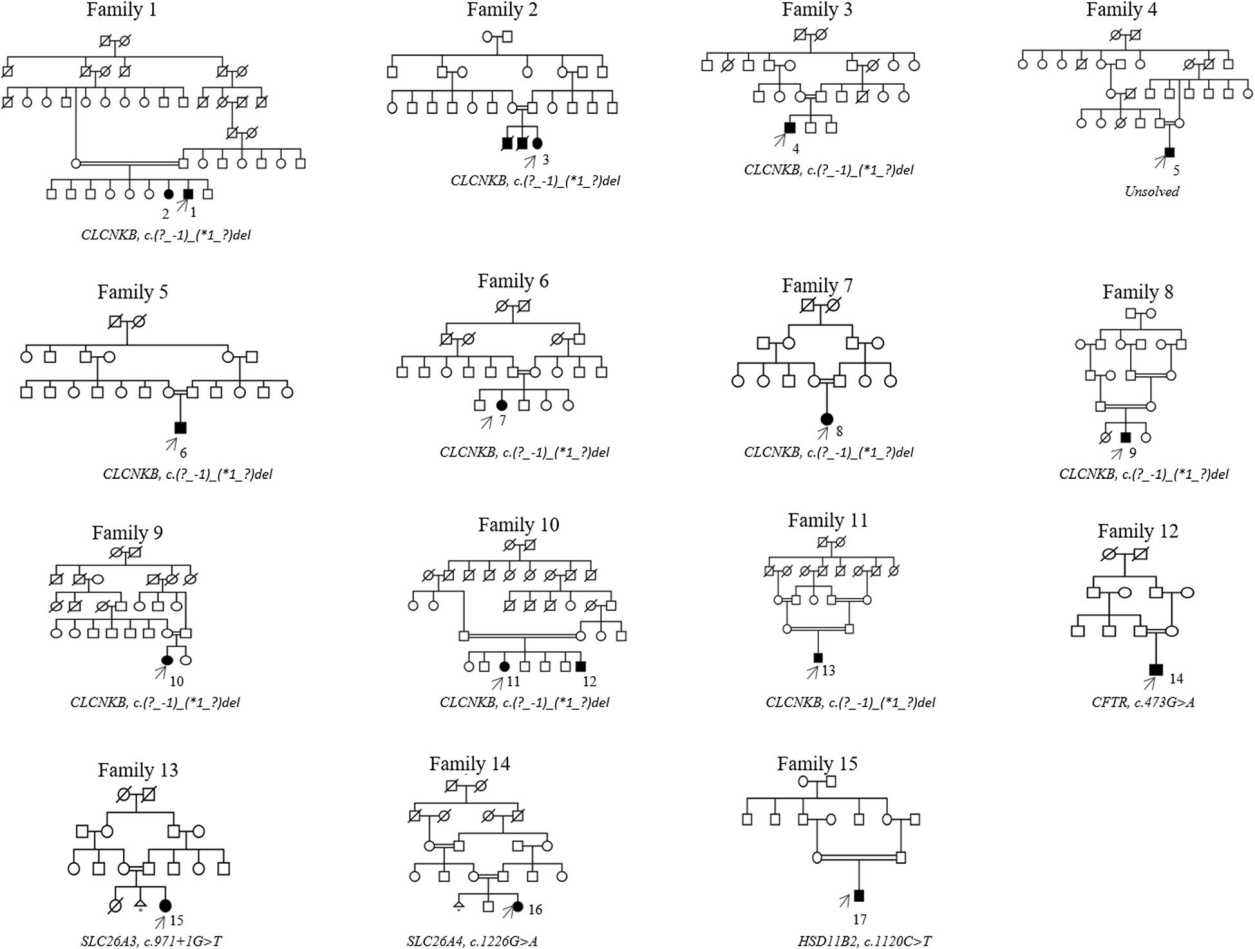
Pedigrees of the 15 families included in this study and identified mutations. Affected individuals included in the study are numbered, arrows indicate the index cases within the study

Pedigrees of all 15 families are shown in Fig. 2, Normal ranges of urinary calcium/creatinine ratio in children is shown in Additional file 1: Figure S2, Sanger sequencing primers can be found in Additional file 1: Figure S3, sequencing results for family 12,13, 14 and 15 are shown in Additional file 1: Figure S4.

## Discussion

In this Iranian cohort, deletion of the entire *CLCNKB* gene was identified as the most common allele causing BS. This is in line with previous findings in other populations where this deletion has also been found to represent the most common BS allele, especially in BS type III. In our cohort, phenotypes resulting from the deletion ranged from type I BS to Gitelman syndrome. All individuals in our cohort originated from the same region in the Southeast of Iran (Baloch ethnicity). It is possible families are remotely related and share a distant common ancestor. Interestingly, phenotypic presentations e.g. with regards to the age at onset of the first symptoms and presentations resembling different BS subtypes were highly variable amongst individuals harboring the identical *CLCNKB* deletion. Intrafamilial phenotypic variability has been previously reported in a very large inbred Bedouin kindred in Northern Israel as well as a Spanish family [23, 30].

**Fig. 3:**
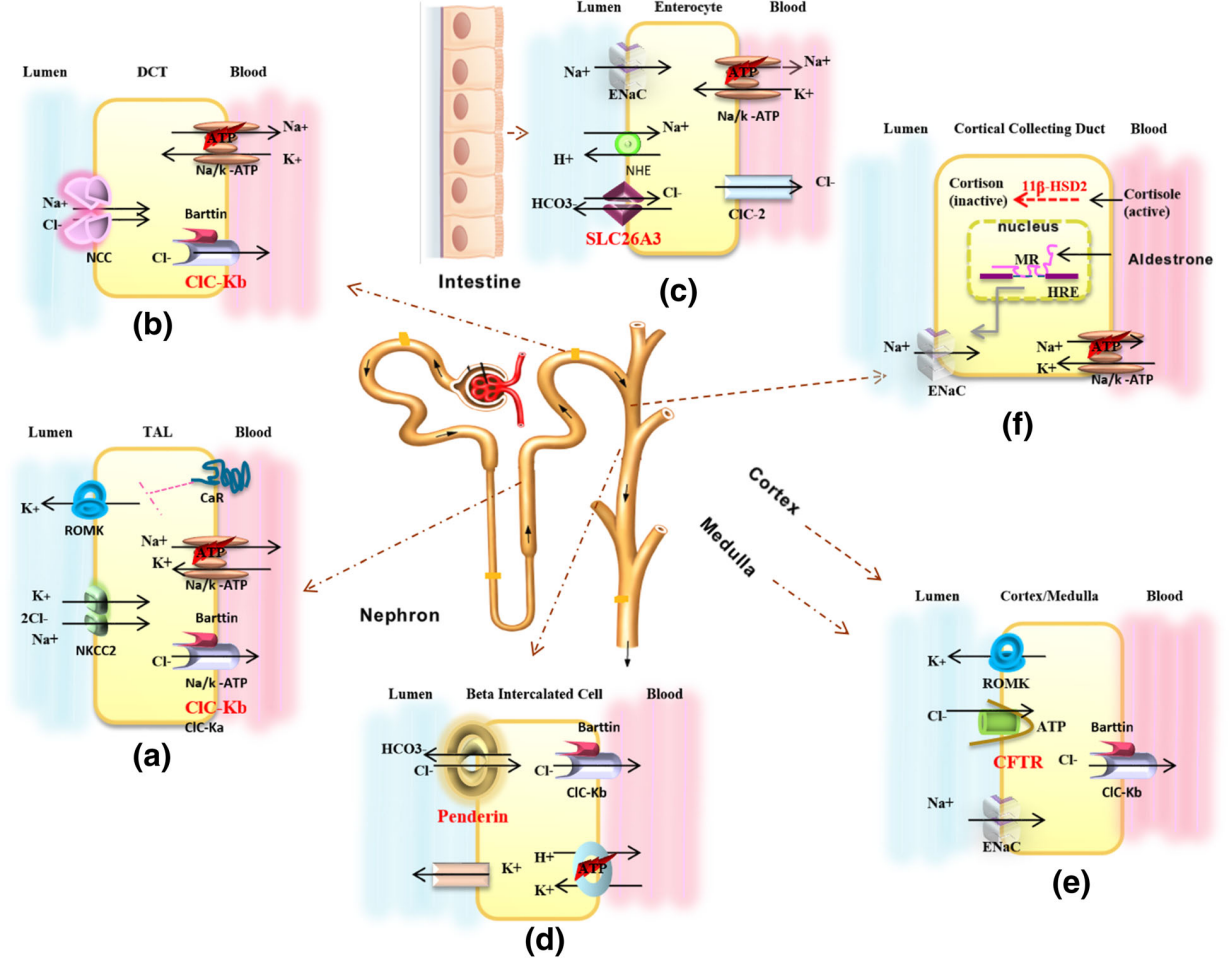
Schematic of localizations and function of ion transporters defective in Bartter Syndrome and Pseudo-Bartter conditions. ClC-kb is mainly found in the thick ascending loop of Henle (TAL), (**a**) and distal tubules (DCT), (**b**) of kidneys, SLC26A3 in the intestine (**c**), Pendrin localizes mainly to renal beta-intercalated cells (**d**), CFTR is found in all nephron segments (**e**), and 11β-HSD2 enzyme in cortical collecting duct (**f**). (**a**) Thick ascending loop of Henle: Luminal NKCC2 enables import of Na+, K+ and Cl- into the cells. K+ flows back to the lumen through ROMK1 channels; Na+ and Cl- are reabsorbed to the blood stream through Na+/K+ ATPase and ClC-kb channels. CASR inhibits the luminal ROMK channel which in turn results in decreased NaCl reabsorption and increased urinary Cl-. (**b**) Distal Tubulus: Cl- transport occurs via the luminal, NCCT and exit to blood by ClC-kb. (**c**) In enterocytes, Cl- absorbed from the intestinal lumen via SLC26A3 and transported to the interstitium by ClC-2. Na+ enters the cell via ENaC channels or Na+/ H+ exchangers and is transported to the interstitium by the Na+/k+ ATPase. (**d**) Penderin participates in urinary bicarbonate excretion with tubular Cl- reabsorption. (**e**) CFTR functions as a Cl- channel and CFTR functions influences other ion channels such as ENaC and ROMK in the cortex and medulla. (**f**) mineralocorticoid aldosteron binds to Mineralcorticoid receptors (MR) which in turn binds to the hormone response elements (HRE) in the nucleus and stimulates increased resorption of Na+ from the urine through transcription of genes involved in ENac and Na+/K+ ATP channels. Simultaneously glucocorticoid cortisol oxidized to inactive cortisone by 11β-HSD2 enzyme

Putatively, the phenotypic differences observed could be caused by non-genetic factors e.g. environmental influences or result from the different genetic background between individuals (multigenic cause). Specifically, genotypic differences with regards to other (chloride) channels and transporters in the nephron resulting in different expression levels or function could also play a role (Fig. 3) [31, 32].

To date, no larger studies have been published about the incidence of clinical misdiagnosis of BS where in fact other conditions are causative for the symptoms. Congenital chloride diarrhea results from loss of function mutations in the *SLC26A3* gene encoding an Cl^−^/HCO_3_^−^ exchanger in the intestine (Fig. 3c). Previous to our report, confounding congenital chloride diarrhea with BS has been reported in 2 additional cases without mentioning the underlying mutations [14, 15]. Potentially, watery diarrhea could be confounded with urine and with increasing dehydration, the amount of diarrhea decreases, making a diagnosis of congenital chloride diarrhea even more difficult. Unlike in BS patients where high urinary Cl^−^ concentration is found, low urinary and high fecal Cl^−^ is detected in congenital chloride diarrhea [33]. Thus, both congenital chloride diarrhea and BS should be considered as differential diagnosis in patients with hypokalemic metabolic alkalosis. Untreated congenital chloride diarrhea can be lethal due to the acute and chronic dehydration and secondary impairment of renal function. Although by KCl or NaCl substitution electrolyte levels can be kept in balance, this does not influence the amount of diarrhea.

Second clinical misdiagnosis of BS in our cohort concerns a case which genetically turned out to represent Pendred syndrome. This syndrome is characterized by severe to mild hearing loss and euthyroid goiter [34]. Diagnosis of BS in this 18 months old was made base on hypokalemic metabolic alkalosis with sensorial deafness without any sign of euthyroid goiter. As goiter manifestation develops usually after the age of 10 years, Pendred syndrome can easily misdiagnosed in infants as our case [35]. Only a handful of patients with SLC26A4 mutations and metabolic alkalosis have been reported in the literature including a 46-year-old Caucasian female with sensorineural deafness and hypothyroidism (Cl^−^: 86 mmol/l, K^+^:1.4 mmol/l, HCO_3_^−^: 45 mmol/l), a child following thiazide therapy (potassium 1.7, chloride 70, sodium 129, HCO3 43.8, base excess + 17.8 mmol/l, pH 7.52), and another 46-year-old woman with sensorineural deafness, hypothyroidism, and profoundly low potassium levels (K^+^:1.4 mmol/l, HCO_3_^−^: 45 mmol/l) [16, 36, 37]. Pendred syndrome is caused by mutations in *SLC26A4* encoding Pendrin which acts as Cl^−^/HCO_3_^−^ exchanger in the inner ear, thyroid and kidney [34]. Under basal conditions, Pendrin mediates acid- base balance through HCO_3_^−^ excretion and Cl^−^ reabsorption in the kidney in the β-intercalated cells of the cortical collecting duct in the kidney (Fig. 3d) [38]. Loss of function in the kidney is usually compensated by the other transporters; therefore, malfunction of Pendrin in the kidney does usually not result in detectable clinical symptoms. However, it is suggested that Pendrin might have an additional adaptive role in eliminating excess bicarbonate in alkalosis conditions [16]. Pendred syndrome therefore should be considered in infants or very young children with hypokalemic alkalosis and sensorial deafness alongside BS.

The third genetic revision of the initial clinical diagnosis in our cohort concerns a case with homozygous *CFTR* missense mutation manifesting as pseudo-BS. *CFTR* is a chloride channel expressed in many organs, including the kidneys. A number of publications have reported to date that in regions with hot climate, patients younger than 2 years with CF may present electrolyte disturbance without any other signs of respiratory and gastrointestinal abnormalities [39]. *CFTR* is expressed in all nephron segments where it is not only involved in Cl^−^ transportation but also regulates other ion channels such as ENaC and ROMK through ATP transport (Fig. 3e) [40]. Several other pathogenic *CFTR* variants such as 3849 + 40A > G, 2.789 + 5 G > A, F311 L, T3381, D110H, S13F, D110E, N1303K and ΔF508 have been associated with hypokalemic metabolic alkalosis [41]. To our knowledge, the c.473G > A mutation has not been reported yet to manifest only with pseudo-BS in the absence of respiratory and gastrointestinal symptoms. However, in hot weather, like in southern Iran, high sweat production rate lead to hypokalemia in sweat and urine through massive NaCl loss and secondary hyperaldosteronism Hypokalemic metabolic alkalosis is observed in both BS and CF patients, but urinary chloride loss in BS syndrome is higher than in CF patients.

In the fourth case with a clinical misdiagnosis of BS, we identified ahomozygous *HSD11B2* mutation, reversing the diagnosis to AME. So far, fewer than 100 AME cases have been reported in the literature. The clinical hallmark of this disorder is hypokalemic metabolic alkalosis with severe childhood- or juvenile-onset hyporeninemic hypertension [41]. Under normal conditions, the mineralocorticoid aldosteron binds to mineralcorticoid receptors (MR) which in turn bind to hormone response elements (HRE) in the nucleus, resulting in transcription of ENac and Na^+^/K^+^ ATP channels in renal collecting duct cells. Simultaneously, the glucocorticoid cortisol which has similar MR affinity as aldosterone is oxidized to the inactive form cortisone by the 11β-HSD2 enzyme (Fig. 3f), inhibiting binding to MR. Under conditions of impaired 11β-HSD2 function, cortisol which is present in 1000–2000 times higher concentrations compared to aldosterone, binds to MR, causing enhanced Na^+ −^reabsorption which in turn leads to the expansion of intravascular fluid, causing hypertension [42]. Diagnosis of BS in the 6 month old patient described here was made based on hypokalemic metabolic alkalosis with low birth weight, failure to thrive and poor growth. Hypertension however only occurs later during the course of AME, thus does not help to differentiate AME from BS in toddlers. [41, 43]. In addition, because the incidence of hypertension is low in children less than three years of age, routine blood pressure monitoring is not recommended unless the patient is at risk for hypertension, facilitating misdiagnosis of AME.

**Fig. 4:**
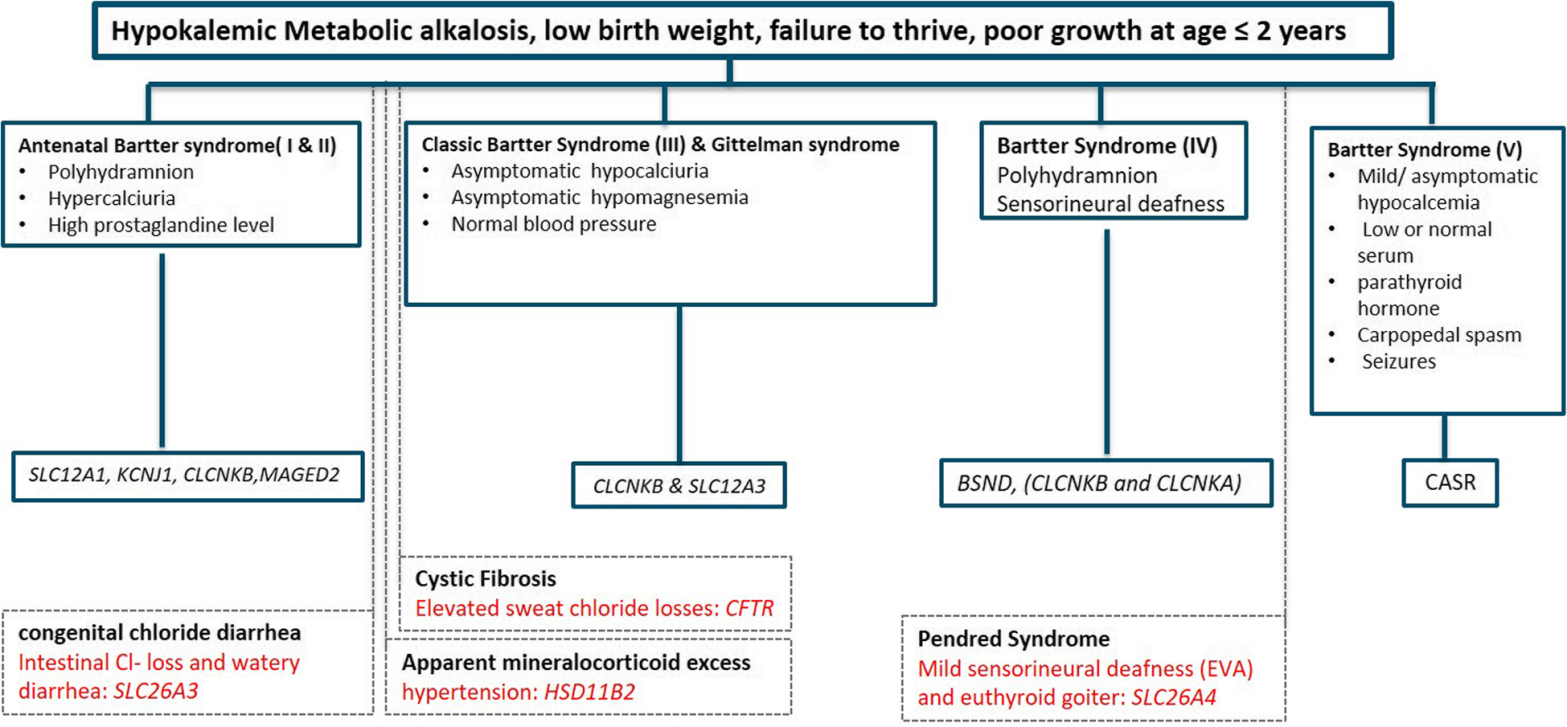
Flow chart for diagnostic investigation of the Bartter syndrome, including genetic analysis. Antenatal Bartter syndrome (I & II) with nephrocalcinosis, polyhydramnion, hypercalciuria and high prostaglandin level characteristics mostly caused by mutations in *SLC12A1, KCNJ1* genes and in rare cases by mutations in *CLCNKB,* or in X-linked cases it caused by mutation in *MAGED2* genes. During follow-up examintation of these patients clinicians should also consider intestinal Cl^−^ loss and watery diarrhea for differential diagnosis from congenital chloride diarrhea which is caused by mutation in *SLC26A3* gene. Classic Bartter syndrome (III) and Gitelman syndrome caused by mutation in *CLCNKB* and *SLC12A3* genes, usually appeared in childhood but in rare cases they could also be present in infants. During follow-up examination of these patients clinicians should also examine hypertension for differential diagnosis from Apparent mineralocorticoid excess which is caused by mutation in *HSD11B2* gene. And also they should scrutinize sweat chloride losses for differential diagnosis from cystic fibrosis. Type IV Bartter syndrome which is accompany with sensorial deafness caused by *BSND* gene or digenic mutation in *CLCNKA* and *CLCNKB* genes. During follow-up examination clinician should consider euthyroid goiter which usually have late onset manifestation for differential diagnosis from Pendred syndrome which is caused by mutation in *SLC26A4* gene. Finally type V Bartter which is caused by mutation in CASR gene characterized by mild or asymptomatic hypocalcemia, low or normal serum parathyroid hormone, carpopedal spasm seizures and also it is associated with dominant phenotype of Bartter syndrome

In summary, clinical misdiagnosis of BS in our cohort was nearly 25%. This suggests a generally high chance for BS misdiagnosis, especially in developing countries where elaborated biochemical analysis is not available due to considerable phenotypic overlap between different rare hypokalemic disorders. Additionally, current clinical classification of antenatal BS, classic BS and Gitelman syndrome is complicated and not always specific. We found *CLCNKB* deletions to cause a broad phenotypic spectrum. A clinical diagnostic algorithm is proposed in Fig. 4 for patients with hypokalemic metabolic alkalosis, low birth weight and failure to thrive within the first 2 years of life. Common biochemical markers related to different types of Bartter syndrome, also watery diarrhea, hypertension, sweat chloride losses, enlarged vestibular aqueducts (EVA) visible in CT-scans and euthyroid goiter should be actively looked out for.

However, NGS approaches now allow time and cost efficient parallel analysis of several (or all) coding genes, offering additional diagnostic possibilities, independent of correct clinical phenotype classification. In this study, WES costs per sample were 250 USD including bioinformatics (Novogene, Hongkong), not including variant filtering performed in-house using the variant files provided by the company. Wider implementation of WES will hopefully result in more precise diagnosis and targeted treatment approaches as well as new genetic counseling opportunities, especially in countries with limited biochemical testing facilities.

## Conclusions

In summary, our data suggests that BS patients negative for the common *CLCNKB* deletion profit from WES and that Pseudo-BS e.g. due to CF, congenital chloride diarrhea, Pendred Syndrome and AME should be considered as differential diagnosis in infants and young children with hypokalemic metabolic alkalosis.

## Methods

### Human research subjects

Consent forms were obtained from all the participants of this study. Ethical approval committee of Mashhad University of medical sciences approved this study (IR.MUMS.REC.1395.534). 17 patients from 15 families referred to our clinics from 2016 to 2018 with diagnosis of BS were included. The basic information and laboratory results of the patients have been summarized in Table 1 and pedigrees have been shown in Fig. 1.

### DNA extraction

Genomic DNA was extracted from whole blood, using standard salting out method. The concentration of DNA was measured by Qubit 2.0 (life technologies, Carlsbad, CA, USA).

### WES

2 microgram DNA from 6 patients out of 17 patients [5, 13–17], and was used for WES, using Illumina HiSeq 2500, Q30 ≥ 80% (Novogene, Hongkong). Exome capture was performed with Agilent SureSelect Human All Exon V6 Kit, sequencing depth was 50× using paired-end sequencing on a HiSeq 2500 Genome Analyzer (Illumina), resulting in sequences of 150 bases from each end of the fragments. UCSC hg19 was used as a reference genome. VarScan version 2.2.5 and MuTec and GATK Somatic Indel Detector were used to detect SNV and InDels, respectively. Data was filtered for MAF < 1% in public control databases such as dbSNP, ExAc and gnomad (gnomAD, http://gnomad.broadinstitute.org). Additionally, variants occurring with MAF > 0.01 in the Iranome (http://www.iranome.ir) were also excluded. The remaining variants were filtered for known disease causing genes first and we prioritized homozygous variants due to the autosomal recessive inheritance pattern of disease and consanguinity. For families without plausible variants left after filtering, BAM files were visually inspected for homozygous CNVs in known disease causing genes related to a hypokalemic metabolic phenotype.

### PCR and sanger sequencing

Conventional PCR was done by Taq polymerase (Roche, Mannheim, Germany) based on manufacture’s instruction to extend the mutated region with specific primer. Also, long range PCR (200 bp-4 kb) was done by AccuPrime™ Taq DNA polymerase system to confirm deletion in *CLCNKB* gene with specific primer according to the following condition, 2.5 μl of 10x AccuPrime™ PCR Buffer II, 0.5 μl of Primer Mix (10 μM each), 1-200 ng of template DNA, 0.5 μl of AccuPrime™ Taq DNA polymerase in total of 25 autoclaved distilled water (initial denaturation at 94 °C for 2 min; followed by 40 cycles of denaturing at 94 °C for 30s, annealing at 65 °C for 30s, and extension at 68 °C for 3 min and final extension at 68 °C for 5 min). Primer sequences are in Table S1. Before Sanger sequencing, PCR products were cleaned by ExoSAP-IT® (USB, Cleveland, Ohio, USA). The cleanup PCR products were bidirectional sequenced using 3730XL DNA analyzer (ABI, Foster City).

### Web resources

Homozygosity-Mapper, http://www.homozygositymapper.org/.

Exome Aggregation Consortium (ExAC), http://exac.broadinstitute.org/.

Genome Aggregation Database (*gnomAD*), http://gnomad.broadinstitute.org/.

dbSNP, http://www.ncbi.nlm.nih.gov/SNP/.

1000 Genomes Project human polymorphism database, http://www.1000genomes.org/.

National Heart, Lung and Blood Institute–Exome Sequencing Project, http://evs.gs.washington.edu/EVS/.

Online Mendelian Inheritance in Man, http://www.omim.org/.

Integrated genome viewer (IGV), http://software.broadinstitute.org/software/igv/.

## Additional file

Additional file 1: **Figure S1.** Aminoacid conservation of CFTR p.Ser158Asn. **Figure S2.** Normal ranges of urinary calcium/creatinine ratio in children. **Figure S3.** Sanger sequencing primers used. **Figure S4.** Sanger traces of identified mutations in non-Bartter-Syndrome genes. (DOCX 648 kb)

## Abbreviations

AME: Apparent mineralocorticoid excess
BS: Bartter Syndrome
BSND: Barttin CLCNK type accessory beta subunit
CASR: Calcium sensing receptor
CFTR: Cystic fibrosis transmembrane conductance regulator
CLCNKB: Chloride voltage-gated channel Kb
DCT: Distal convoluted tubule
HRE: Hormone Response Elements
KCNJ1: potassium voltage-gated channel subfamily J member 1
MAGED2: MAGE family member D2
MR: Mineralcorticoid receptors
NCC: renal thiazide-sensitive NaCl cotransporter
NKCC2: Na-K-Cl cotransporter
PTC: Proximal tubular cells
ROMK: Renal outer medullary potassium channel
SLC12A1: Solute Carrier Family 12 Member 1
SLC26A3: Solute Carrier Family 26 Member 3
SLC26A4: Solute Carrier Family 26 Member 4
TAL: Thick ascending limb
WES: Whole exome sequencing
