# Supplementary material for: Mimicry and well known genetic friends: molecular diagnosis in an Iranian cohort of suspected Bartter syndrome and proposition of an algorithm for clinical differential diagnosis
Source: Orphanet J Rare Dis. 2019 Feb 13;14:41. doi: 10.1186/s13023-018-0981-5 (PMC6375149; doi:10.1186/s13023-018-0981-5)
Supplement: Supplementary file 1 — Figure S1. Aminoacid conservation of CFTR p.Ser158Asn. Figure S2. Normal ranges of urinary calcium/creatinine ratio in children. Figure S3. Sanger sequencing primers used. Figure S4. Sanger traces of identified mutations in non-Bartter-Syndrome genes. (DOCX 648 kb) [file 13023_2018_981_MOESM1_ESM.docx]

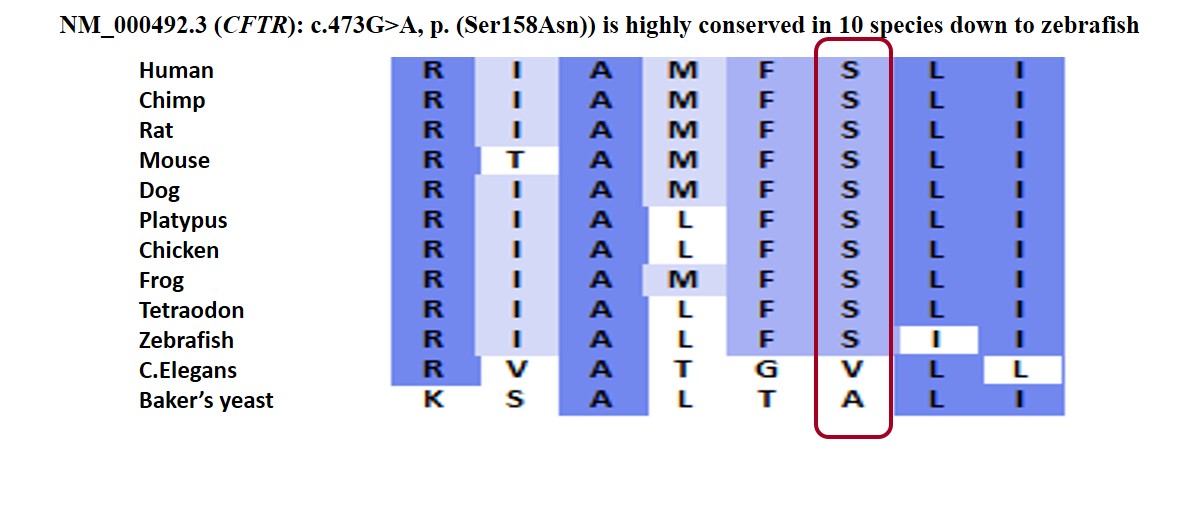
**Figure S1. Aminoacid conservation of CFTR p.Ser158Asn**


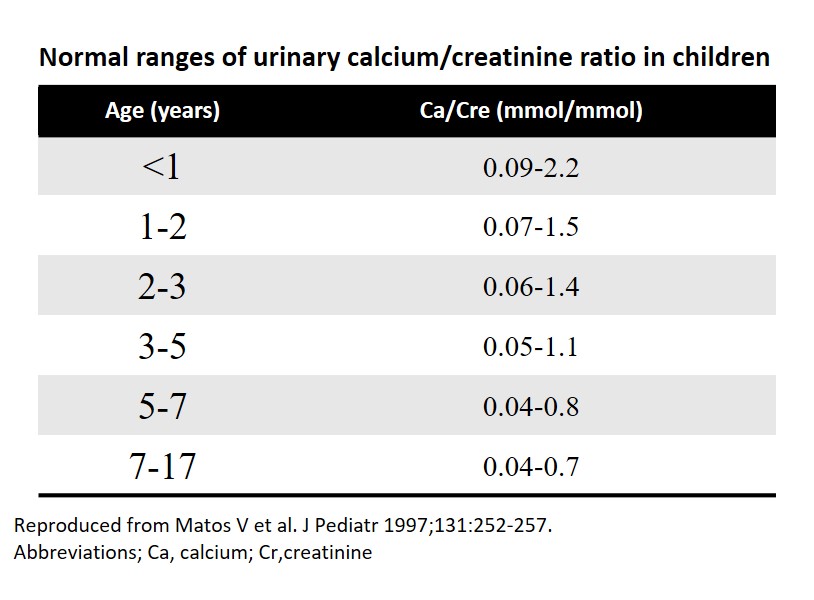


**Figure S2. Normal ranges of urinary calcium/creatinine ratio in children**

| **primer** | **Sequence** | **Tm** |
| --- | --- | --- |
| *CLCNKB_EXON9_F* | GTCAGGCTCTGGGCTCATGTC | 65 |
| *CLCNKB_EXON14_R* | GACTCAGCCTGAGGTGGGCAC | 65 |
| *CFTR_F* | TCTTGTGTTGAAATTCTCAGGGTA | 59 |
| *CFTR_R* | TGAGTCATCTTAACAGGAAACCAT | 59 |
| *SLC26A3_F* | GCAGTTTTCGGGCATAATGT | 60 |
| *SLC26A3_R* | ATCCTCTCACCTTGGCCTCT | 60 |
| *SLC26A4_F* | GGTATGGCGTCCAAACTCC | 60 |
| *SLC26A4_R* | GGTGAGGGAGTGGAACAAGA | 60 |
| *HSD11B2_F* | CACTTGCATGGGCAGTTCCTGC | 59 |
| *HSD11B2_R* | AGGAACCAAGGGCTCACGGA | 59 |

**Figure S3. Sanger sequencing primers used.**

**
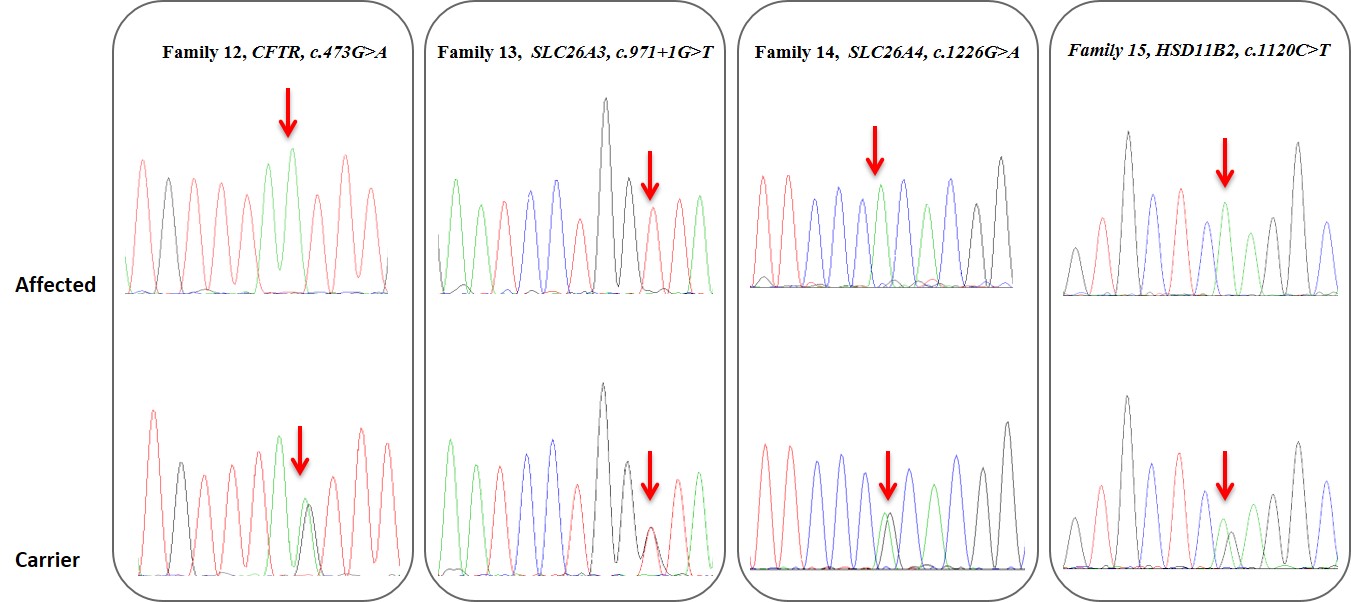
**

**Figure S4. Sanger traces of identified mutations in non-Bartter-Syndrome genes**
